# The Prevention of Fire

**Published:** 1913-02-01

**Authors:** W. J. Dobbie

**Affiliations:** Physician-in-Chief, Toronto Free Hospital, Weston.


### THE PREVENTION OF FIRE.

By Dr. W. J. DOBBIE, Physician-in-Chief, Toronto Free Hospital, Weston.

[Read before the Canadian Hospital Association.]

(*Concluded from page* 464.)

On the subject of fire drill there seems to be a great difference of opinions. Not only are there as many hospitals without fire drill as with it, but the nature of the drill differs essentially in many particulars in the hospitals where it prevails. I think, however, that it may safely be said that in every hospital the matter of what to do in case of fire should be seriously considered so that some definite plan of action may be thoroughly understood by those whose duty it is to guard the lives of the sick and the helpless who have for the time being been entrusted to their care.

### Fire Drill and its Difficulties.

It would be unwise, however, as I take it, to go to the extreme of saying, or even recommending, that an actual drill should be practised, because in many of the smaller institutions such a procedure is a practical impossibility, and would if attempted be little short of a farce. The paramount difficulties are two in number: (1) That employees change too frequently to enable results to be obtained; (2) Very few hospitals have on their staff a person competent to organise and direct such a drill. In these cases it is much better that something less pretentious but more within their reach be attempted. A plan of action carefully thought out by the superintendent, in which a few definite duties are assigned to different members of the staff, will produce much better results in time of need. In our own experience we never had an actual test or drill. Each individual knew, however, where his or her post of duty was, and the result was satisfactory to a degree beyond all expectation.

Each hospital superintendent must decide what is the best thing to be done in his or her hospital. Give each person one clearly defined duty to perform and keep that prominently before them. My own method is to have typewritten cards tacked on the door of each person's bedroom. That supplies the information; perhaps once a month, as each employee comes to the cashier for his wages, I question him as to his duty in case of fire. If he cannot answer satisfactorily he cannot draw his pay. Perhaps each month one or two are sent back, but the same man is never caught a second time.

Some of the larger institutions, however, deem it wise to have an actual test. This makes it necessary, of course, to have a false alarm of some kind. This in itself I consider a serious objection to this system, because no matter how much care is taken to warn patients that false alarms for fire are likely to be given, there is also a certain degree of excitement and anxiety produced which is, to say the least, very undesirable. For those, however, who wish to have an actual test all that is necessary is: (1) To systematise the duties of all persons employed, and (2) to provide a suitable alarm.

### A Scheme of Duties.

In arranging a scheme of duties a plan has been described by Richard H. Townley, Superintendent of Lincoln Hospital and Home, as follows: "An efficient fire drill, whether for a hospital of fifty beds or one thousand beds, can be made with very little trouble. Each person has a number, no personality, so that if Susan Brown, one of the laundresses—No. 17 for instance, or 127—is discharged and her place is filled by somebody else, her successor takes that number, and she is given a card when she is employed by the hospital telling her what her duties are at fire drill. Then at the fire drill either yourself or your assistant see that every station, every duty, is actually carried out. It is not necessary to start the water at all times, but be ready to start it."

The great objections I see to this system are: (1) That every time a new employee is engaged his number must be assigned him and his card of instruction given him; and (2) that employees are assigned duties in accordance with the work performed, and not according to the location of their sleeping quarters, which is, I think, a most important provision to make.

These are obviated by having the duties determined by the room occupied, as I have previously indicated.

### Night Watchmen.

My inquiries have revealed the fact that in hospitals of one hundred beds or less a night watchman is rarely employed. In hospitals of more than one hundred beds one is usually employed. Further, in those hospitals in which a watchman is employed there are only a very few cases in which he has not other duties to perform, such as those of night fireman, night orderly, night porter, etc.

I believe that every hospital could with advantage employ a night watchman. But I am equally certain that his services will be of little value unless checked by an automatic clock. Portable clocks which the watchman can carry with him are to be had. These record on a paper disc the time at which the watchman reports at different stations. They are absolutely sure in their records, and the watchman must report at each station in order to punch the paper disc, and the time and station are accurately recorded in every case.

The safe removal of patients may, as I take it, be considered one of the most important measures. Care should be taken that every ward is manned by a sufficient number of helpers to care for all the patients without loss of time.

In our experience the helpless patients were much less trouble than those able to help themselves. The former were picked up bodily and carried to a place of safety. The latter became excited, and in some cases stubborn, and required a deal of watching to prevent accidents or loss of life through their confusion as to routes, etc. Stretchers of special design should be provided for such patients as cannot well be moved otherwise. Horse-blanket pins in each ward would appear to be useful, a patient being pinned in a blanket so that two people can carry him conveniently. The military method of carrying a patient by means of a single bearer should be learned by all nurses, as by that method a person weighing 115 lb. or 120 lb. can carry another of much greater weight.

A number of good ideas have been received which I take the liberty of incorporating. Among these are: (1) Attention to chimneys and flues. (2) Fire doors. (3) Fire shutters. (4) Abolition of parlour matches in hospitals. (5) Danger from Christmas decorations and fireworks. (6) Cellars and closets to be kept free from collections of waste. (7) Daily inspections. (8) Instruction given to staff by member of fire brigade. (9) Oily cloths should be burned immediately and not put into drawers or cupboards. (10) Special alarm direct to city fire hall. (11) Visit furnace room and kitchens often.

In conclusion it may be said that so many suggestions have been received that it has been quite impossible to make acknowledgment individually. I desire, however, to express my appreciation of the great assistance thus received, without which indeed this paper would have been impossible. I trust, moreover, that our combined effort may prove of assistance to many, and that superintendents and boards of trustees may everywhere be incited to assure themselves of the safety of the sick and the helpless who have been entrusted to their care.

